# Prevalence and Persistence of Post-COVID-19 Condition After Critical Care: 32-Month Follow-Up

**DOI:** 10.3390/jcm15020711

**Published:** 2026-01-15

**Authors:** Alicia Ávila Nieto, Paulo Infante, Francisco Javier Barca Durán

**Affiliations:** 1 Doctoral School, University of Valladolid, 47011 Valladolid, Spain; 2 School of Science and Technology, Department of Mathematics, Research Center in Mathematics and Applications, University of Évora, 7000-671 Évora, Portugal; pinfante@uevora.pt; 3 Human Anatomy Department, Nursing and Occupational Therapy School, University of Extremadura, 10003 Cáceres, Spain; jbarca@unex.es

**Keywords:** post-COVID-19 condition, critical COVID-19, prevalence, follow-up studies

## Abstract

**Background/Objectives:** Post-COVID-19 condition (PCC) remains poorly characterized beyond two years, particularly among intensive care unit (ICU) survivors. We aimed to describe the prevalence, persistence, and late consequences of PCC up to 32 months after discharge in an ICU cohort. **Methods:** This single-center longitudinal cohort included 170 adults with confirmed SARS-CoV-2 infection admitted to an ICU in Cáceres (Spain) between March 2020 and March 2021. 94 survivors entered follow-up at discharge and 3, 6, 12, 18, 24, and 32 months. PCC manifestations were grouped into five organ system domains (respiratory, cardiovascular, renal, infectious, and musculoskeletal/neuromuscular) and recorded only when supported by clinician-confirmed diagnoses or diagnostic tests. Prevalence at each visit, persistence, and new onset of manifestations between 3 and 6 months, and the cumulative incidence of new chronic diseases between 18 and 32 months were estimated with 95% confidence intervals. **Results:** Any PCC manifestation was almost universal at discharge (96.8% [95% CI, 91.1–98.9]) and remained high at 12 months (85.2% [95% CI, 76.3–91.2]), declining to 48.6% at 24 months and 25.7% at 32 months. Respiratory manifestations predominated early and were largely resolved by 32 months, whereas musculoskeletal/neuromuscular involvement remained relatively stable. From 18 to 32 months, 36.5% (95% CI, 26.4–47.9) of survivors developed at least one chronic condition, most frequently cardiovascular disease (14.9% [95% CI, 8.5–24.7]). **Conclusions:** Long-term PCC manifestations and incident chronic diseases are common among ICU COVID-19 survivors, underscoring the need for prolonged follow-up and post-ICU care.

## 1. Introduction

The COVID-19 crisis has been a global public health emergency with no precedent in this century. During the pandemic, restrictive measures and lockdowns shaped lifestyle changes for most people around the world. At first, the disease was mostly described as a viral respiratory infection, with a high potential to progress to atypical severe pneumonia and, eventually, cause death [1]. Intensive care units (ICUs) across the globe started gradually to fill with patients requiring extremely high levels of mechanical and systemic support [2,3]. Researchers soon realized that COVID-19 was not merely an acute infection. Reports of long-lasting post-acute sequelae started to unveil a new and not yet well-understood condition, affecting not only the lungs but also many other body systems [4]. Early cohort studies already described persistent organ impairment with cardiovascular, respiratory, renal, hepatic, endocrine, or immune involvement, finding single-organ manifestations in 70% of the cohort and multi-organ dysfunction in 29% at four months after acute infection [5]. The underlying cause appeared to be a complex interplay of mechanisms, including persistent immune activation, endothelial and microvascular injury, autonomic and neuroinflammatory disturbances, and, in some patients, an amplified thromboinflammatory response. These interlinked processes were profoundly disabling with substantial individual and societal consequences [6,7]. Several large systematic reviews and meta-analyses have now characterized sequelae after COVID-19 acute infection across multiple domains, including respiratory, cardiovascular, musculoskeletal, and neuropsychiatric sequelae. However, many of these syntheses rely predominantly on patient-reported outcomes and symptom checklists, which may limit comparability with studies that use clinician-confirmed organ-specific diagnoses [8,9,10,11,12,13]. Additional findings on the phenomenon suggested that many patients did not feel fully back to their baseline health despite the absence of overt post-infection fibrosis or resting hypoxia on routine evaluation. Several cohorts have reported that persistent symptoms such as dyspnea are not consistently explained by standard lung function or imaging indices of acute disease severity, although more advanced techniques, including hyperpolarized xenon magnetic resonance imaging (MRI) and perfusion single-photon emission computed tomography/computed tomography (SPECT/CT), have revealed subtle microvascular and ventilation–perfusion abnormalities that may contribute to ongoing respiratory complaints. In this sense, the relationship between acute phase severity, detectable structural damage, and long-term symptom burden appears to be complex rather than linear [14,15,16,17]. Nevertheless, COVID-19 critical care survivors face a much more complex reality. The comprehensive rehabilitation needed after hospitalization and the inadequate access to post-ICU follow-up were placing a burden on these patients and their families, whereas the impact of their long-term sequelae was being understudied and soon gained attention among the research community [18]. The available literature describing post-COVID specifically in ICU survivors is sparse and often concentrates on self-reported psychological symptoms and quality of life [19,20]. The multisystem involvement that characterizes hospital and ICU admission has been implicated in the mechanism responsible for prolonged physical consequences of COVID-19, a condition now referred to as post-COVID-19 condition (PCC) [21]. The term PCC is the terminology adopted by the WHO and is used in this manuscript to refer to persistent, multisystem sequelae after SARS-CoV-2 infection, broadly overlapping with what other authors and agencies have termed “long COVID” or “post-COVID-19 syndrome” [22,23]. Recent long-term studies have found a high prevalence and persistence of PCC across severe hospitalized COVID-19 patients (38.1% after two years), yet the multi-organ involvement and specific causes remain to be better understood. This highlights the multisystem nature of the syndrome, demanding a holistic and interprofessional approach [24]. Coordinated structured management of the condition has already been described, and healthcare professionals are encouraged to collaborate within multidisciplinary teams to ensure effective patient care [25]. The World Health Organization (WHO) has also stated the need for consistent and longer-term data on PCC, especially in low-income and rural areas, with limited access to healthcare services [23]. The aim of this study was to determine the prevalence and persistence of PCC over a 32-month period following hospital discharge in ICU COVID-19 survivors in a rural setting.

## 2. Materials and Methods

This single-center longitudinal cohort study included 170 adults with confirmed SARS-CoV-2 infection who were admitted to the ICU at the University Hospital of Cáceres (Spain) during the first and second pandemic waves (2020–2021). This hospital is the main referral center in the province and one of the largest in the region, hosting the province’s primary ICU; therefore, it was chosen for participant recruitment. Cáceres is widely recognized as a rural area, both within Spain and across Europe, so the findings of this study should be interpreted in the context of a rural population. The study size was determined by including all consecutive ICU admissions with confirmed COVID-19 during the study period; a priori sample size calculation was not performed. Participants were included according to the following criteria: (1) admission to the ICU between March 2020 and March 2021; (2) confirmed diagnosis of COVID-19; (3) willingness to participate in the study; and (4) signed informed consent by the patient or, if the patient was unable to provide consent, by a legally authorized representative.

A total of 170 patients met these criteria and were recruited during hospitalization. Of these, 76 patients died during the acute phase. The remaining 94 survivors were discharged and entered the follow-up program. During follow-up, one additional death occurred before the 3-month visit and two more before month 6. Only one patient was lost to follow-up within the first six months. Sixteen more patients were lost after 12 months, leaving 74 survivors with complete assessments through 32 months (Figure S1). STROBE guidelines were followed to report this research.

### 2.1. Data Collection

Clinical and hospitalization data (including medical history, vital signs, treatments, and other relevant variables) were retrieved from the hospital’s electronic medical records and entered into a REDCap database. To streamline data entry, a standardized data collection form was developed based on the study variable list. Follow-up data were obtained from hospital records, on-site visits, and structured telephone interviews at each follow-up time point beginning three months after hospital discharge. Baseline demographic characteristics, exposure history, comorbidities, and symptom information were recorded at hospital admission. Sequential Organ Failure Assessment (SOFA) scores were documented at ICU admission as part of acute-phase severity assessment. Evaluations were conducted at discharge and at 3, 6, 12, 18, 24, and 32 months post-discharge (M3, M6, M12, M18, M24, and M32, respectively), from March 2020 through December 2023. “Lost to follow-up” was defined as surviving participants who did not attend scheduled visits and could not be reached for further data collection; deaths were recorded separately. All outcomes were analyzed using available-case denominators for each time point. Clinical manifestations were categorized into five PCC-related organ system domains (respiratory, cardiovascular, renal, infectious, and musculoskeletal/neuromuscular). Domains were not mutually exclusive; a given patient could contribute to more than one domain at a given visit. “Any PCC” was defined as ≥1 positive domain.

Respiratory manifestations included exertional or resting dyspnea, cough, chest tightness, wheeze, and pleuritic pain, supported by abnormal clinical examination or objective findings such as oxygen desaturation, abnormal chest imaging, or reduced spirometric parameters (including DLCO) and, when present, referral to pulmonary rehabilitation. Cardiovascular manifestations were recorded when chest pain, palpitations, exertional intolerance, orthostatic symptoms, or syncope were judged compatible with cardiovascular dysfunction and supported by ECG abnormalities, elevated cardiac biomarkers, echocardiographic findings, documented arrhythmias, or autonomic dysfunction. Infectious manifestations were limited to microbiologically, serologically, or molecularly confirmed infections (e.g., urinary tract infection, bacterial pneumonia, herpesvirus reactivation) diagnosed after discharge, with compatible clinical findings documented by a physician. Renal manifestations included clinically verified kidney dysfunction and/or laboratory abnormalities such as reduced eGFR, albuminuria, hematuria, or a diagnosis of chronic kidney disease or incomplete recovery from COVID-associated acute kidney injury. Musculoskeletal/neuromuscular manifestations comprised clinician-confirmed myalgia, arthralgia, proximal muscle weakness, myopathy (including critical illness myopathy), neuropathic symptoms (e.g., paresthesias, sensory deficits), or chronic fatigue/post-exertional malaise when these were documented as neuromuscular or functional impairment on clinical evaluation. Manifestations were recorded only when supported by diagnostic test results or clinician-confirmed diagnoses; patient-reported symptoms alone were not included. For each domain and visit, manifestations were coded as binary variables (present/absent) according to the study data dictionary.

### 2.2. Data Analysis

All analyses were performed using R version 4.2.2 (R Foundation for Statistical Computing, Vienna, Austria). The primary aim of this study was descriptive (prevalence over time, short-term trajectories between structured clinical visits, and incident diagnoses during extended follow-up). Given the modest sample size, attrition at later time points, and low event counts in several domains, multivariable predictive modeling was not pursued to avoid overfitting and unstable estimates. The five prespecified organ system domains were analyzed individually and combined into an overall measure of any PCC, defined as the presence of ≥1 positive domain at a given visit. For each time point, prevalence was estimated as $\hat{p}$ *=* x/n with 95% confidence intervals (CIs) calculated using the Wilson method. To assess potential attrition bias, we compared baseline demographic and clinical characteristics between patients with complete and incomplete follow-up (age, sex, comorbidities, and admission SOFA score). Missing values were handled using an available-case approach at each time point. No imputation procedures were applied.

Domain trajectory analysis was defined as the presence of a given organ system domain at hospital discharge and at each follow-up visit among participants with data available at both time points. Proportions were reported with Wilson 95% CIs. Persistence and new-onset were evaluated specifically between the structured clinical visits at 3 and 6 months: persistence was defined as a positive value of the domain at both M3 and M6, and new-onset at 6 months as the appearance of the domain at M6 among patients without that domain at M3. New-onset chronic conditions identified during long-term follow-up were summarized and defined as first clinician-documented diagnoses between 18 and 32 months in participants with no previous history of the corresponding disease. These conditions were analyzed separately from PCC organ system manifestations, and we did not attempt to attribute causality to SARS-CoV-2 infection at the individual level. Thus, symptomatic PCC domains describe visit-based organ involvement, whereas “new chronic disease” reflects newly coded long-term diagnoses during late follow-up. All endpoints were harmonized as binary (0/1) variables according to the study data dictionary. The cumulative incidence of new diagnoses of organ-specific chronic diseases (cardiovascular, respiratory, renal, neurologic, metabolic, endocrine, rheumatologic, musculoskeletal, hematologic, oncologic, and psychological) was described both in aggregate and by system.

## 3. Results

### 3.1. Study Population

Demographics, comorbidities, and exposure to SARS-CoV-2 are summarized in Table 1. Women accounted for a smaller proportion of ICU admissions with confirmed COVID-19 than men (32.9% vs. 67.1%), while the overall mean age was 65.73 (SD = 11.99) years. Overall, 59.2% of patients admitted to the ICU had a diagnosis of hypertension (HTN), 27.8% had diabetes mellitus (DM), and 25.4% were obese.

75.6% of COVID-19-positive patients admitted to the ICU presented with fever on hospital arrival, and 79.2% reported shortness of breath. Cough and fatigue were also common at admission (55.4% and 42.4%, respectively). The SOFA score at ICU admission indicated moderate organ dysfunction, with a median of 4.0 (IQR 3.0–5.0).

Baseline age, sex, comorbidities, and admission SOFA score were similar between patients with complete and incomplete follow-up, suggesting a low risk of systematic attrition.

### 3.2. Prevalence and Persistence

**Figure 1:**
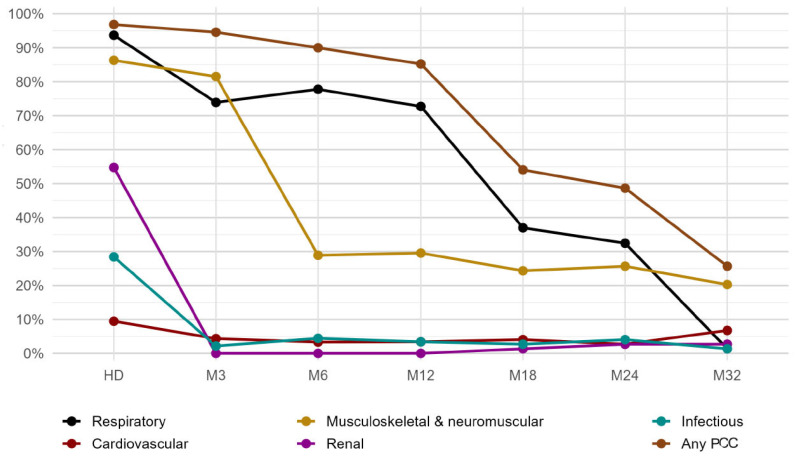
Prevalence of PCC at every time point.

When analyzing the prevalence of PCC over time, the data showed that the presence of manifestations in any organ system was almost universal at hospital discharge (96.8% [95% CI, 91.1–98.9%]) and remained high through 12 months (85.2% [95% CI, 76.3–91.2%]), declining substantially after the first year. PCC-related organ system domains were present in 48.6% [95% CI, 37.6–59.8%] at 24 months and 25.7% [95% CI, 17.1–36.7%] at 32 months. Respiratory manifestations were the most prevalent throughout the follow-up period, followed by musculoskeletal and neuromuscular manifestations, except for month 3, when this second domain showed the highest prevalence, and month 32, when respiratory outcomes appeared to resolve almost completely, while the prevalence of musculoskeletal and neuromuscular manifestations remained relatively high (20.3% [95% CI, 12.7–30.8%]). This domain seemed to be the most stable in prevalence from month 6 onwards, with little improvement over time (Figure 1).

Renal and infectious domains were the next most prevalent at discharge (54.7% [95% CI, 44.7–64.4%] and 28.4% [95% CI, 20.3–38.2%], respectively), exhibiting a clear recovery after month 3 and becoming almost anecdotal until month 32 (2.7% [95% CI, 0.7–9.3%] renal manifestations and 1.4% [95% CI, 0.2–7.3%] infectious manifestations). In the case of the cardiovascular domain, a low prevalence was observed at discharge (9.5% [95% CI, 5.1–17.0%]), which decreased over time to 2.7% [95% CI, 0.7–9.3%] at month 24. Nevertheless, it became the third most prevalent domain at month 32 (6.8% [95% CI, 2.9–14.9%]), although wide confidence intervals reflect the small number of events.

#### Persistence and New-Onset PCC Manifestations Between 3 and 6 Months

**Figure 2:**
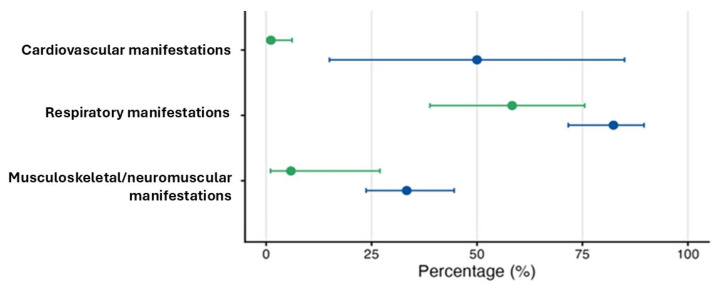
Evolution of PCC manifestations between 3 and 6 months after ICU admission.

When restricting to patients with paired M3–M6 data, there were marked differences in the evolution of manifestations across organ system domains (Figure 2). Respiratory involvement showed the highest persistence: 55/66 patients with respiratory manifestations at M3 still had them at M6 (83.3% [95% CI, 73.0–90.4%]), and more than half of those without respiratory manifestations at M3 developed new respiratory involvement by M6 (14/23; 60.9% [95% CI, 40.1–78.5%]).

Despite their high prevalence at M3, musculoskeletal/neuromuscular manifestations exhibited lower persistence over time: only 25/72 patients with musculoskeletal/neuromuscular involvement at M3 continued to have it at M6 (34.7% [95% CI, 24.8–45.9%]), and incident musculoskeletal/neuromuscular involvement at M6 was infrequent (1/17; 5.9% [95% CI, 1.0–27.0%]). Cardiovascular manifestations were rare but, when present, often persisted (2/4; 50.0% [95% CI, 18.8–81.2]), whereas infectious manifestations were infrequent at M3 and did not persist in any patient. No renal manifestations were observed at either assessment.

Together, these patterns highlight that respiratory manifestations tended both to persist and to develop again between M3 and M6 among ICU survivors.

For each domain, the blue markers and lines show persistence (proportion of patients with the manifestation at M3 who also had it at M6), and the green markers show new onset at 6 months (proportion of patients without the manifestation at M3 who developed it at M6). Proportions are presented with 95% Wilson confidence intervals. Renal and infectious manifestations were too rare to estimate persistence or new onset with precision.

**Figure 3:**
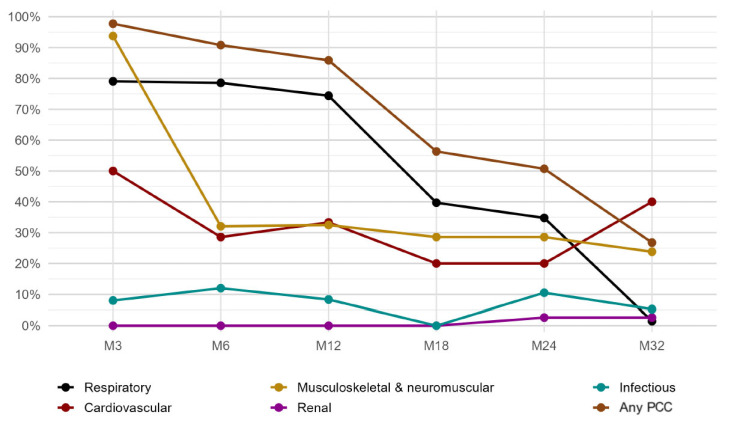
Longitudinal trajectory of PCC at every time point.

As presented in Figure 3, the trajectory (within-patient paired comparisons of manifestation presence between hospital discharge and each time point) of overall PCC followed a similar path to prevalence. Respiratory manifestations (the most prevalent within-subject evaluation from month 6 to 24, except for M3) largely resolved by month 32 (1.4% [95% CI, 0.3–7.8%]). Although cardiovascular manifestations suggested a late increase at the same time point (40.0% [95% CI, 11.8–76.9%]), the low numbers and the extremely wide CIs make this interpretation uncertain. The musculoskeletal and neuromuscular domain improved markedly after 6 months but also remained prevalent at 32 months after discharge (23.8% [95% CI, 15.0–35.6%]).

Overall, these longitudinal patterns highlight the predominance and persistence of respiratory manifestations up to month 32; however, they tended to resolve thereafter. In contrast, the prevalence and evolution of musculoskeletal and neuromuscular manifestations, although lower over time, seemed to remain stable and prevalent at month 32.

### 3.3. Cumulative Incidence of New Chronic Diseases

**Figure 4:**
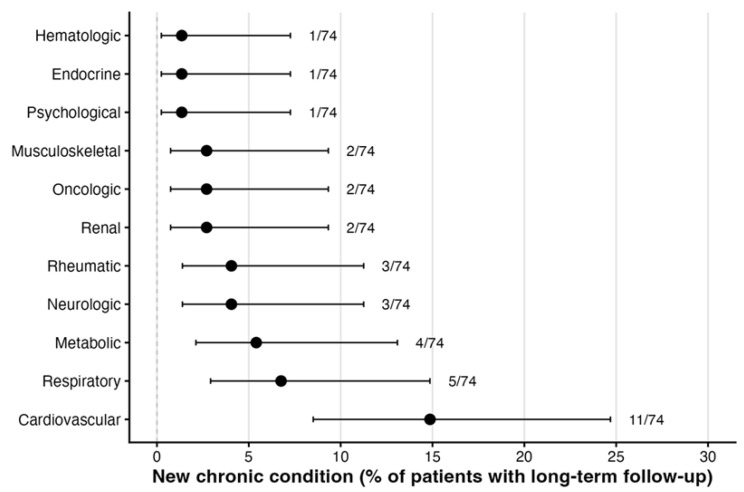
Cumulative incidence of new chronic diseases between 18 and 32 months after ICU discharge among survivors with complete long-term follow-up (*n* = 74).

Among the 74 patients with complete information on new diagnoses during long-term follow-up, 27 developed at least one new chronic condition from 18 to 32 months after discharge (36.5% [95% CI, 26.4–47.9%]), indicating that new chronic conditions were relatively common despite the decline in PCC prevalence (Table 2, Figure 4). New cardiovascular disease was the most frequent, affecting 11/74 patients (14.9% [95% CI, 8.5–24.7%]) and occurring roughly two to three times more often than any other single system. New respiratory and metabolic diseases were recorded in 5/74 (6.8% [95% CI, 2.9–14.9%]) and 4/74 patients (5.4% [95% CI, 2.1–13.1%]), respectively. New neurologic and rheumatic diseases each occurred in 3/74 patients (4.1% [95% CI, 1.4–11.3%]), while renal, oncologic, and musculoskeletal diseases were identified in 2/74 patients each (2.7% [95% CI, 0.7–9.3%]). Psychological, endocrine, and hematologic conditions were rare, with 1/74 case per domain (1.4% [95% CI, 0.2–7.3%]). Confidence intervals are wide, reflecting small numbers, but the pattern consistently suggests that cardiovascular diagnoses account for a substantial proportion of the incident chronic disease burden.

Points represent estimated proportions with 95% Wilson confidence intervals. Numbers on the right indicate *n/N* for each diagnostic category.

## 4. Discussion

This cohort of ICU patients with COVID-19 from the first and second pandemic waves was characterized by the predominance of male patients (67.1%), advanced age (65.73 [SD = 11.99]), and heavy metabolic and cardiovascular comorbidity (59.2% HTN, 27.8% DM, 25.4% obesity). Shortness of breath (79.2%), cough (55.4%), and fatigue (42.4%) were the most common symptoms at hospital admission. These baseline conditions are similar to those described in previous studies [23]. They generally had moderate organ dysfunction on their arrival to the ICU (SOFA 4.0 [IQR 3.0–5.0]), consistent with the hospitalized patient profile of the early pandemic [26]. After 24 months, almost 50% (48.6% [95% CI, 37.6–59.8%]) of patients presented manifestations compatible with PCC. This high prevalence of PCC manifestations in ICU patients has also been described in studies with larger cohorts [27]. Prevalence of any PCC manifestation remained high (85.2% [95% CI, 76.3–91.2%]) during the first year but declined markedly thereafter, especially in the cardiovascular, renal, and infectious domains. This longitudinal improvement has been previously reported and is associated with the end of the early post-acute phase. However, almost a quarter of the population continued to experience manifestations after 32 months [28]. While respiratory manifestations were the most prevalent and persistent over time, with almost complete resolution by 32 months, the musculoskeletal and neuromuscular domain remained steadily prevalent from the sixth month, similar to the findings of the current literature [29]. In a recently published meta-analysis on musculoskeletal symptoms, a prevalence of around 25% was reported, with no significant changes over time [9]. These results contribute to a more comprehensive view of the longitudinal evolution of these manifestations, already described in the literature as the longest-lasting sequelae [10].

Across all five PCC-related organ system domains evaluated in this study, cardiovascular involvement is the only one to show a marked difference between the paths of prevalence and trajectory, suggesting a potential deterioration of manifestations after 32 months. However, low numbers and wide CIs prevent these results from supporting firm interpretations and should be interpreted with caution. 14.9% (95% CI, 8.5–24.7%) of patients had developed a new chronic cardiovascular disease after 32 months, supporting current evidence on the matter [11].

36.5% (95% CI, 26.4–47.9%) of patients had developed a chronic disease by 32 months. Cardiovascular disease was predominant, followed by respiratory disease (6.8% [95% CI, 2.9–14.9%]), metabolic disease (5.4% [95% CI, 2.1–13.1%]), and neurologic disease (4.1% [95% CI, 1.4–11.3%]). Recent findings describe similar cardiovascular sequelae, with almost 10% of incident chronic heart failure (CHF) associated with the previous severity of the acute infection with SARS-CoV-2 [30]. Although absolute numbers were modest, these incident diagnoses highlight the potential for COVID-19–related critical illness to contribute to new multimorbidity beyond the acute phase. These data represent additional evidence regarding healthcare utilization and burden, giving population-level evidence of the increased demand and need for in-depth and personalized follow-up of patients with PCC, especially ICU survivors [31]. Further work in understanding and addressing this condition is essential for public health. Recent studies have reported that about 60% of patients with PCC have unmet care needs, and more than 25% are currently unable to work due to post-COVID sequelae [32].

The prevalence and persistence of PCC in ICU survivors detailed in this study are consistent with the evidence of long-term sequelae from 2-year follow-up studies in non-hospitalized patients [33]. However, the latest literature with broader population samples suggests nearly five times higher (or even more) odds of PCC (OR 4.86 [95% CI, 2.83–8.35]) in patients with severe acute symptoms compared to those with mild symptoms [29,34]. Furthermore, the real extent of PCC manifestations and symptoms is probably higher than suggested in this study. There are three reasons for this: first, PCC was only recorded when there was clear diagnostic evidence of the event, leaving behind many only self-reported symptoms; second, the population studied consists of survivors of severe COVID-19, so there is probable survivor bias, meaning the healthiest and most resilient people entered the follow-up study; and third, neurocognitive and psychological symptoms were not included in the observations, which are usually the most quantified among COVID-19 patients. This last choice is not arbitrary. Although a growing body of work has described post-COVID sequelae across respiratory, cardiovascular, musculoskeletal, and neuropsychiatric domains [9,10,11,12,17], a substantial proportion of the early and most visible literature has focused on neurologic and mental health outcomes, often based primarily on patient-reported measures. In the present study, we intentionally restricted our primary outcomes to clinician-confirmed physical organ system manifestations to avoid diluting the signal from less frequently investigated but clinically relevant physical sequelae, and to provide a complementary perspective to the existing patient-reported outcome measurements (PROM)-driven evidence [12,35,36,37,38,39,40]. Taken together, these factors likely led to conservative estimates of PCC prevalence and persistence in this cohort.

### 4.1. Strengths

This research summarizes the evolution over time of PCC in COVID-19 critical care survivors from hospital discharge to 32 months. The long follow-up period of the study, with several time point observations, along with the accuracy of the data collected, makes these findings a strong contribution to the field. A recently published meta-analysis has highlighted the importance of addressing gaps in the literature, such as overestimation of PCC due to the inclusion of mental health symptoms in the definition, as these may have very diverse causes, such as unreported previous medical history, hospitalization, socioenvironmental factors during the pandemic, or the effects of long-term treatment. The need for long follow-up observations was also identified to provide valuable information about long-term symptom trajectories beyond the post-acute phase. Additionally, several studies use self-reported outcomes, which are also subject to recall and response bias [32]. Missingness patterns also shaped the analytical strategy. Baseline data were nearly complete, and follow-up completeness between 3 and 6 months was very high (96%), minimizing the risk of attrition bias. However, missingness increased in late follow-up, particularly for respiratory outcomes, where the operational definitions differed from those in M3/M6 (Figure S2). As described in the Methods, we used an available-case approach, excluding variables with structurally missing data, and avoiding imputing symptoms whose surveillance varied across time. Importantly, patients with incomplete follow-up were similar in age, comorbidities, and admission severity to those with complete follow-up, suggesting that loss to follow-up is unlikely to have introduced major selection bias.

### 4.2. Limitations

The interpretation of late complications and new chronic diseases must distinguish between symptom persistence and disease evolution. The cumulative incidence of new chronic diseases after 32 months likely reflects a combination of critical illness, progression of pre-existing comorbidities, and general health deterioration, rather than a simple linear continuation of acute post-COVID complications. At the same time, some of these incident chronic conditions, particularly in the cardiovascular and respiratory systems, may represent delayed or downstream sequelae of SARS-CoV-2 infection rather than entirely unrelated disease. However, in the absence of a control cohort and formal adjudication, we cannot determine causality at the individual level, so we report these diagnoses descriptively as new chronic conditions while acknowledging that they may overlap conceptually with late PCC manifestations. Similarly, the presence of respiratory or neuromuscular symptoms at late follow-up cannot be directly linked to earlier symptoms without repeated structured assessment. Recognizing these uncertainties reinforces the importance of standardized follow-up protocols in post-ICU cohorts and highlights the need for continuous, harmonized monitoring to accurately map long-term recovery trajectories.

Because this cohort includes ICU survivors, some long-term manifestations may overlap with post-intensive care syndrome (PICS) and other sequelae of critical illness (e.g., deconditioning, ICU-acquired weakness, neurocognitive impairment), rather than being specific to SARS-CoV-2 infection. Without a non-COVID ICU control group and without detailed ICU exposure metrics (e.g., ventilation duration, ICU length of stay), attribution to COVID-specific mechanisms should be made cautiously.

Although predictive factor analyses could have been clinically valuable, the combination of modest sample size, attrition, and rare domain-specific endpoints would have made multivariable prediction prone to overfitting; larger multicenter cohorts are required for robust model development and validation.

The modest sample and the multimorbidity and advanced age background may introduce bias to the long-term effects of PCC, and results should be interpreted with caution. The lack of homogeneous diagnostic tools and a control group also restricts comparability. Missing data on other organ systems are another limitation to consider. Persistence analyses were deliberately restricted to the 3- and 6-month assessments, which were the only time points with harmonized symptom definitions, standardized clinical evaluation, and fully paired patient-level data. These features allowed us to quantify true persistence (continuation of symptoms from M3 to M6) and new-onset manifestations at M6 with appropriate denominators and 95% confidence intervals. Consequently, complications recorded at 18–32 months cannot be interpreted as longitudinal persistence of earlier symptoms, nor can their onset be causally linked to M3/M6 findings. This distinction is essential to avoid overinterpreting long-term trajectories.

Importantly, our operational definition of PCC is conservative: manifestations were recorded only when supported by clinician-confirmed diagnoses or diagnostic tests. Therefore, the reported prevalence and persistence primarily reflect organ-specific, clinically substantiated sequelae, and should not be interpreted as capturing the full spectrum of PCC symptoms (e.g., subjective fatigue, headache, cognitive complaints) typically reported using symptom checklists or PROMs, limiting direct comparability with PROM-driven definitions. Also, SARS-CoV-2 reinfections during follow-up were not systematically captured. Given the duration of observation and evolving community transmission, some participants may have experienced one or more reinfections that could have contributed to symptom persistence, new manifestations, or incident chronic diagnoses. Finally, our assessment of organ system sequelae was limited to tests performed as part of routine clinical care. Very specialized investigations and tissue-based diagnostics were not applied systematically, so some cardiovascular, respiratory, or other organ sequelae may have remained undetected. Accordingly, when we describe manifestations as having “resolved,” this refers to the absence of clinically overt, test-confirmed disease with the methods used in this study and does not exclude the possibility of persisting subclinical pathology.

## 5. Conclusions

PCC is a complex multisystem condition affecting different organs from patient to patient. The evolution of prevalence is intense during the first year, easing gradually thereafter. However, for some patients, symptoms may persist beyond 32 months and, eventually, become chronic. Further studies with larger and more heterogeneous samples and specific clinical outcomes are needed. Exploring the development of chronic diseases and longer-term consequences for individuals and health systems would provide valuable insights into new care plans and management strategies.

## Figures and Tables

**Table 1:** Baseline characteristics of the study population.

Variable	N	*n* (%)	Median [IQR] Mean (SD)
Sex	170	Female 56 (32.9%)	
		Male 114 (67.1%)	
Age (years)	170		Median [IQR]: 67.0 [59.0, 75.0]
			Mean (SD): 65.73 (11.99)
BMI (kg/m^2^)	162		Median [IQR]: 28.19 [26.04, 31.14]
			Mean (SD): 28.77 (4.42)
ICU SOFA score	120		Median [IQR]: 4.00 [3.00, 5.00]
			Mean (SD): 4.58 (1.84)
Nursing home resident (on admission)	169	8 (4.7%)	
Health-care worker	167	10 (6.0%)	
Known COVID-19 contact	114	57 (50.0%)	
Travel history before admission	154	12 (7.8%)	
Influenza vaccination	156	66 (42.3%)	
Pneumococcal vaccination	155	5 (3.2%)	
Smoker	103	7 (6.8%)	
Alcohol use disorder	156	3 (1.9%)	
Hypertension	169	100 (59.2%)	
Travel history before admission	154	12 (7.8%)	
Influenza vaccination	156	66 (42.3%)	
Pneumococcal vaccination	155	5 (3.2%)	
Smoker	103	7 (6.8%)	
Alcohol use disorder	156	3 (1.9%)	
Hypertension	169	100 (59.2%)	
Diabetes mellitus	169	47 (27.8%)	
Obesity	169	43 (25.4%)	
Chronic kidney disease	169	10 (5.9%)	
Chronic heart disease	169	18 (10.7%)	
Asthma	169	15 (8.9%)	
Chronic obstructive pulmonary disease (COPD)	169	11 (6.5%)	
Interstitial lung disease (ILD)	169	4 (2.4%)	
Cognitive disorder	169	14 (8.3%)	
Rheumatic disease	169	13 (7.7%)	
Hematologic disorder	169	12 (7.1%)	
Malignant neoplasm	169	9 (5.3%)	
Immunosuppression	169	6 (3.6%)	
Organ transplant	169	1 (0.6%)	
Endocrine disorder	169	21 (12.4%)	
Metabolic disorder	169	53 (31.4%)	
Initial symptoms at hospital admission			
Fever	168	127 (75.6%)	
Cough	166	92 (55.4%)	
Dyspnea (shortness of breath)	168	133 (79.2%)	
Chest pain	168	25 (14.9%)	
Fatigue	165	70 (42.4%)	
Headache	168	17 (10.1%)	
Diarrhea	169	34 (20.1%)	
Anosmia	134	20 (14.9%)	
Ageusia	134	20 (14.9%)	

Values are presented as *n* (%) or median [IQR]/mean (SD), as indicated. Abbreviations: ICU, intensive care unit; SOFA, Sequential Organ Failure Assessment; COPD, chronic obstructive pulmonary disease; ILD, interstitial lung disease; IQR, interquartile range; SD, standard deviation.

**Table 2:** Cumulative incidence of new chronic diseases.

New Chronic Disease	*N/n*	% [95% CI]
New overall disease	74/27	36.5% [26.4–47.9%]
New cardiovascular disease	74/11	14.9% [8.5–24.7%]
New endocrine disease	74/1	1.4% [0.2–7.3%]
New hematologic disease	74/1	1.4% [0.2–7.3%]
New metabolic disease	74/4	5.4% [2.1–13.1%]
New musculoskeletal disease	74/2	2.7% [0.7–9.3%]
New neurologic disease	74/3	4.1% [1.4–11.3%]
New oncologic disease	74/2	2.7% [0.7–9.3%]
New psychological disease	74/1	1.4% [0.2–7.3%]
New renal disease	74/2	2.7% [0.7–9.3%]
New respiratory disease	74/5	6.8% [2.9–14.9%]
New rheumatic disease	74/3	4.1% [1.4–11.3%]

Values are presented as *N/n* and percentage with 95% confidence intervals (CIs). N, total number of patients with complete follow-up; *n*, number of patients with the new chronic disease in each category. The new overall disease includes patients with at least one new chronic condition between 18 and 32 months.

## Data Availability

The data presented in this study are available upon request from the corresponding author due to privacy and ethical reasons.

## Supplementary Materials

The following supporting information can be downloaded at: https://www.mdpi.com/article/10.3390/jcm15020711/s1, Figure S1: Patient flowchart, Figure S2: Missing data (%) across all variables included in the PCC and healthcare-use analyses.

## Abbreviations

The following abbreviations are used in this manuscript:

BMI	Body mass index
CHF	Chronic heart failure
CI	Confidence interval
COPD	Chronic obstructive pulmonary disease
COVID-19	Coronavirus disease 2019
DLCO	Diffusing capacity of the lung for carbon monoxide
DM	Diabetes mellitus
HTN	Hypertension
ICU	Intensive care unit
ILD	Interstitial lung disease
IQR	Interquartile range
MRI	Magnetic resonance imaging
OR	Odds ratio
PICS	Post-intensive care syndrome
PROMs	Patient-reported outcome measures
REDCap	Research Electronic Data Capture
SARS-CoV-2	Severe acute respiratory syndrome coronavirus 2
SD	Standard deviation
SOFA	Sequential Organ Failure Assessment
SPECT/CT	Single-photon emission computed tomography/computed tomography
WHO	World Health Organization
