# Supplementary figures and images for: Prevalence and Persistence of Post-COVID-19 Condition After Critical Care: 32-Month Follow-Up
Source: J Clin Med. 2026 Jan 15;15(2):711. doi: 10.3390/jcm15020711 (PMC12842475; doi:10.3390/jcm15020711)

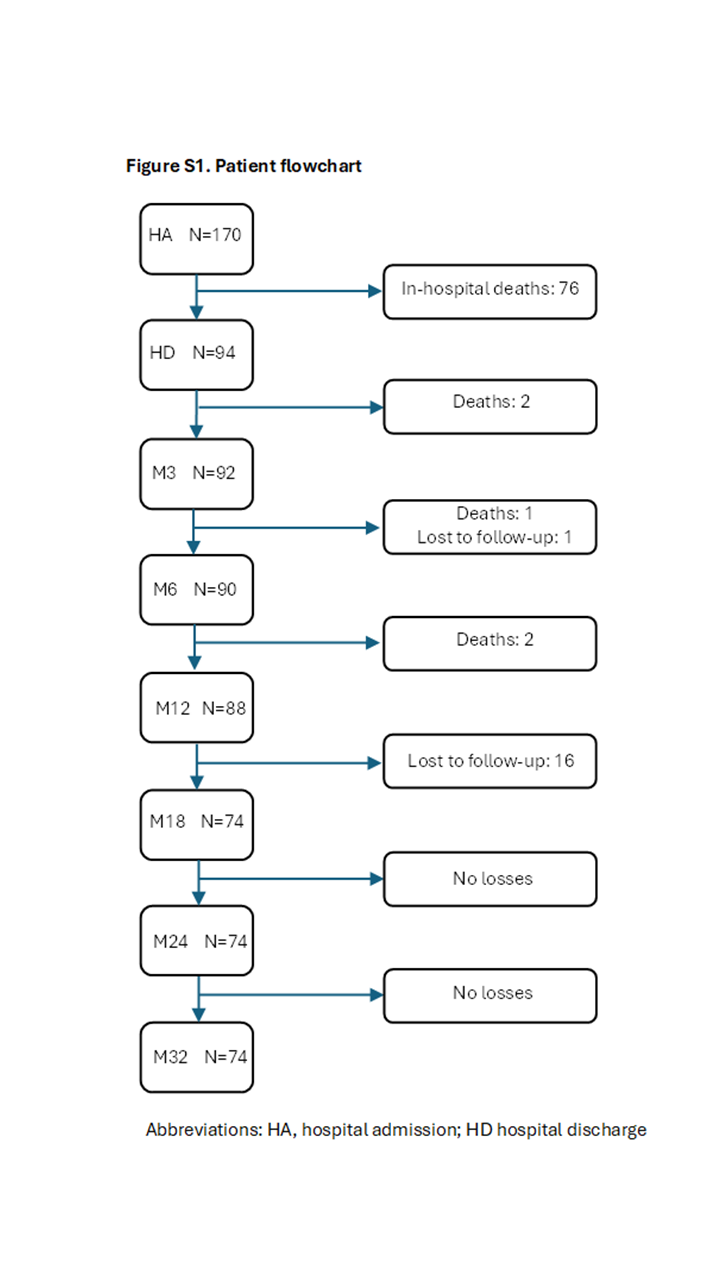

Supplement: Supplementary file 1 [file jcm-15-00711-s001.zip › Figure S1. Patient flowchart.png]

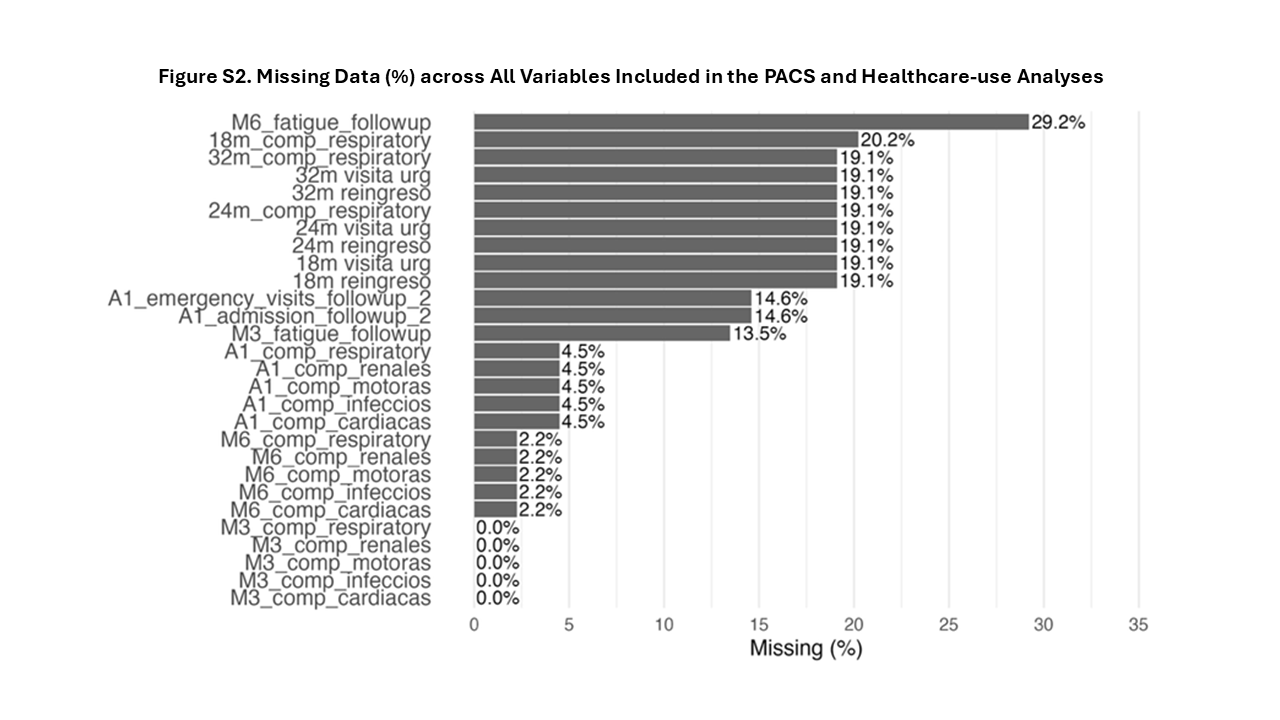

Supplement: Supplementary file 1 [file jcm-15-00711-s001.zip › Figure S2 Missing Data.png]
